# Case Report: Effective and Safe Treatment With Certolizumab Pegol in Pregnant Patients With Cogan’s Syndrome: A Report of Three Pregnancies in Two Patients

**DOI:** 10.3389/fimmu.2020.616992

**Published:** 2021-01-18

**Authors:** Nils Venhoff, Jens Thiel, Markus A. Schramm, Ilona Jandova, Reinhard E. Voll, Cornelia Glaser

**Affiliations:** Department of Rheumatology and Clinical Immunology, Medical Center, Faculty of Medicine, University of Freiburg, Freiburg im Breisgau, Germany

**Keywords:** Cogan’s syndrome, pregnancy, vasculitis, TNF-α inhibitor, Certolizumab pegol

## Abstract

Cogan’s syndrome is a rare autoimmune disease characterized by ocular inflammation and audiovestibular manifestations. Treatment consists of systemic glucocorticoids and other immunosuppressive agents including methotrexate, cyclophosphamide and TNF-α-inhibitors. Due to potential ovarian or fetal toxicity immunosuppressive treatment options are limited during pregnancies. Thus far there is a paucity of reports on pregnancies in Cogan’s syndrome. With minimal transplacental transfer, Certolizumab pegol is considered to be safe for the use in pregnant patients with underlying inflammatory diseases. However, there is no literature on the use of this TNF-α-inhibitor in Cogan’s syndrome in general and especially during gestation. Here we report three pregnancies in two Cogan’s Syndrome-patients treated with Certolizumab pegol. Treatment with Certolizumab pegol was effective and well tolerated in patients with Cogan’s syndrome and seems to be a safe treatment option during pregnancy.

## Introduction

Cogan’s syndrome (CS) is a very rare chronic autoimmune disease characterized by ocular inflammation and audiovestibular symptoms including tinnitus, vertigo and hearing loss (1). It mainly affects young adults without gender predominance (2). Standard treatment consists of systemic glucocorticoids that may be combined with glucocorticoid-sparing agents like methotrexate, azathioprine, cyclosporine, cyclophosphamide or tumor necrosis factor-α (TNF-α) inhibitors (2–4). Management of inflammatory diseases in general is particularly challenging in female patients of reproductive age. On the one hand, adequate disease control at conception and during pregnancy is crucial to ensure maternal and fetal health, on the other hand, available treatment options are limited because of potential ovarian or fetal toxicity. Therefore, treatment with cytotoxic or teratogenic substances like methotrexate or cyclophosphamide should be stopped months before conception. Furthermore, TNF-α inhibitors are often discontinued after the first trimester of pregnancy to limit placental transfer of the drug to the fetus (5). Monoclonal anti-TNF-α-antibodies of IgG1 isotype are actively transported *via* the neonatal fragment crystallizable (Fc) receptor during the second and third trimester) (6). Certolizumab pegol (CZP) is a pegylated Fab fragment of a humanized anti-TNF-α-antibody, approved for the treatment of rheumatoid arthritis and other inflammatory autoimmune diseases. Because of the lacking Fc fragment CZP does not bind to the neonatal Fc receptor and is not actively transferred across the placenta (6). The CRIB study in pregnant women receiving CZP for approved indications showed no quantifiable CZP concentrations in the neonates at time of delivery and during follow-up, indicating zero to minimal placental transfer or fetal exposure during the third trimester (7). Accordingly, CZP is considered safe for the use in pregnant women (5). Cumulatively, only eight pregnancies in six female patients with CS have been described in literature, with no reports existing on the use of biological agents, e.g., CZP (8–13). Here we report the first three pregnancies in two Caucasian patients with CS who received CZP treatment in standard dose with subcutaneous injections of 200 mg every other week. Therapy in both patients was started after a risk-benefit analysis, shared decision making and written informed consent.

## Case Descriptions

### Patient A

At the age of 27, patient (A) experienced several episodes of steroid-sensitive hearing loss accompanied by vertigo and tinnitus, as well as recurrent bilateral conjunctivitis and keratitis (**Table 1**). The patient’s right-sided deafness was treated with a cochlear implant. No further disease manifestations were detectable. The initial immunosuppressive treatment consisted of glucocorticoids in combination with azathioprine, which was later switched to methotrexate due to adverse drug reactions. Additionally, topical treatment with 5% dexpanthenol eye and nose ointment (Bepanthen^®^) and eye drops with hyaluronic acid for dry eyes were used during episodes of keratitis. Upon incomplete therapeutic response, the treatment regimen was supplemented by adalimumab, a human recombinant IgG1 monoclonal antibody directed against TNF-α, resulting in rapid clinical response with good tolerability. Latent tuberculosis was ruled out by chest X-ray and interferon-gamma release assay (IGRA) prior to initiation of adalimumab. Due to a planned pregnancy methotrexate was paused and adalimumab was replaced by CZP in combination with low-dose prednisolone six months before conception. The slightly overweight patient had stopped smoking years before; a mild hypothyroidism was adequately treated. Prior to conception and throughout pregnancy CS was in clinical remission, and serum C-reactive protein (CRP) concentrations were normal or only slightly increased. Screening for antinuclear antibodies (ANA), antiphospholipid antibodies (APA), rheumatoid factor (RF), anti-neutrophil cytoplasmic antibodies (ANCA) was negative at time of initial diagnosis and during follow-ups. Serum concentrations of complement factors C3 and C4 as well as immunoglobulins IgG, IgA and IgM were within normal ranges. Results of tone audiometric monitoring remained stable during follow-up. The patient experienced no complications during pregnancy and delivered a healthy girl by spontaneous vaginal delivery at gestational week 38 after a percentile appropriate intrauterine development. Adherence to CZP was excellent during pregnancy and the prednisolone dose was continuously kept ≤5 mg/day. To prevent loss of bone mineral density 1000 IE of vitamin D3 were substituted daily. CZP-treatment was well tolerated without local reactions at the injection site or other clinically relevant adverse events. Neither the child nor the mother had any postpartum complications. After two years without relevant CS activity, under continued combination therapy of CZP and low dose prednisolone, the patient became pregnant again and gave birth to a healthy boy after 40 unremarkable gestational weeks (**Table 2**). Both children were formula-fed, reached all developmental milestones, and did not develop any serious infections or malignancies during the current follow-up time of five years.

**Table 1:** Patients‘ characteristics and treatment information.

		Patient A	Patient B
Age at time of diagnosis (y)		28	30
Origin		Caucasian / Europe	Caucasian/Europe
Medical history		Hypothyroidism	No other diseases or comorbidities
Family history		Inconspicuous	Inconspicuous
Previous pregnancies		None	One pregnancy with an uncomplicated course and a healthy child
Smoking status		Former smoker	Non-smoker
Body weight; Body mass index (BMI)		69 kg ; 26 kg/m²	55 kg; 20.7 kg/m²
**Clinical manifestations**			
Vestibulo-auditory manifestations		bilateral hearing impairment, unilateral hearing loss (right ear), bilateral tinnitus, vertigo	bilateral hearing impairment, unilateral hearing loss (left ear), bilateral tinnitus, vertigo
Eye manifestations		Bilateral interstitial keratitis Bilateral conjunctivitis	Bilateral conjunctivitis
General symptoms		fever, weight loss, arthralgia	subfebrile temperature, fatigue, myalgia
Other physical examination including neurological status		unremarkable	unremarkable
**C-reactive protein serum concentration as biomarker for disease activity**			
CRP before CZP		28.3 mg/L	6 mg/L
CRP within the first 3 months of CZP		Normalization to values <5 mg/L	Normalization to values <5 mg/L
CRP during follow-up under CZP		At normal dose <5 mg/L, After dose reduction <10 mg/L, After treatment cessation >10 mg/L	Continuously <5 mg/L
**Treatment before start of CZP**			
Topical treatment		5% Dexpanthenol eye and nose ointment (Bepanthen®), Eye drops with hyaluronic acid for dry eyes during episodes of keratitis	Intratympanic steroid injection, 5% Dexpanthenol eye and nose ointment (Bepanthen®), Eye drops with hyaluronic acid for dry eyes during episodes of conjunctivitis
Prednisolone treatment			
	intravenous bolus	PRED 3× 250 mg	PRED 3× 250 mg
	initial oral dose	PRED 60 mg/day	PRED 15 mg/day
	long term dose	PRED ≥ 10 mg/day	0
	treatment duration	32 months	11 months
Synthetic DMARDs		AZA 100 mg/day MTX 20 mg/week	AZA 100 mg/day
Biological DMARDs		ADA 40 mg EOW	ADA 40 mg EOW

ADA, adalimumab; AZA, azathioprine; BMI, body mass index; CRP, c-reactive protein; CZP, certolizumab pegol; DMARDs, disease modifying antirheumatic drugs; EOW, every other week; IGRA, interferon-gamma release assay; MTX, methotrexate; PRED, prednisone; y, years.

**Table 2:** Disease activity, course of pregnancy and nursing period.

	Patient A		Patient B
**Pregnancies**			
Prior pregnancies	None		One pregnancy
	**1^st^ pregnancy**	**2^nd^ pregnancy**	**3^rd^ pregnancy**
CS activity at conception	complete remission	complete remission	complete remission
age at delivery (y)	32	35	33
pregnancy duration (gw)	38	40	41 + 3
Mode of delivery	spontaneous vaginal in labor	spontaneous vaginal in labor	spontaneous vaginal in labor
Child’s condition at birth	healthy	healthy	healthy
APGAR score	10/10	9/10	10 / 10
Umbilical cord pH	7.25	7.29	7.22
Neonatal weight (g)	3270	3740	399
Neonatal length (cm)	54	54	57
Head circumference (cm)	35	33.5	36.5
Intrauterine development	percentile-appropriate	percentile-appropriate	percentile-appropriate
Postpartum complications	None	None	none
**Treatment during pregnancy and nursing period**			
1^st^ trimester	CZP, PRED 5mg	CZP, PRED 4mg	CZP
2^nd^ trimester	CZP, PRED 4mg	CZP, PRED 4mg	CZP
3^rd^ trimester	CZP, PRED 4mg	CZP, PRED 4mg	CZP
Breast feeding	no breast feeding	no breast feeding	yes (CZP continued)
Follow up after birth (m)	59	29	28
Total duration of CZP treatment (months)	74		41

APGAR, **A**ppearance, **P**ulse, **G**rimace, **A**ctivity, **R**espiration Score; cm, centimeters; CS, Cogan’s syndrome; CZP, certolizumab pegol; g, gram; gw, gestational weeks; m, months; PRED, prednisone; y, years.

In total, the patient was continuously treated with CZP for more than six years. Between her two pregnancies, the patient tried to lower the CZP dose at least twice by extending the application interval to three weeks. As these extensions of the treatment interval resulted in no signs of clinical activity but increased serum CRP concentrations, the CZP standard dosing regimen was implemented again. A complete cessation of CZP treatment more than a year after her second pregnancy led to rising CRP serum concentrations up to 20 mg/L, accompanied by fatigue and mild hearing impairments. Immediately after re-initiated administration of CZP 200 mg every other week, in combination with oral prednisolone 10mg/day for one week and consecutive tapering, CRP levels were normal again and clinical symptoms disappeared without any permanent damage.

### Patient B

Three months after the delivery of her first child, the thirty-year-old patient B experienced several episodes of mild conjunctivitis of both eyes and bilateral hearing impairment with tinnitus, vertigo and subsequent unilateral hearing loss (**Table 1**). Myalgias were accompanied by an increase in serum concentrations of creatine kinase and CRP. No further disease manifestations were detectable by extensive physical examination including a detailed neurological assessment. Screening for autoantibodies (ANA, APA, RF, ANCA) was negative, and serum concentrations of C3, C4 and immunoglobulins were within normal range. The conjunctivitis with dry eyes was initially treated topically with 5% dexpanthenol eye and nose ointment (Bepanthen^®^) and eye drops with hyaluronic acid. The ear manifestations required stronger immunosuppressive treatment with intratympanic steroid installation and systemic prednisolone treatment in combination with azathioprine, which subsequently had to be stopped because of hepatic side effects. Second-line treatment with TNF-α blocking agent adalimumab was effective and resulted in normalization of CRP levels, however, was switched to CZP because of the patient’s desire to have children. After an unremarkable pregnancy with normal fetal development, she delivered a healthy boy in the 42^nd^ gestational week (**Table 2**). Under continuation of CZP, her child was breastfed and displayed normal development within the first two years without any notable infections. Since initiation of treatment with TNF-α-inhibitors the patient was in complete, relapse-free remission. The patient continuously presented with normal CRP values and even showed an improvement in tone audiometry of the inner ear hearing threshold by 15–20 decibel hearing level compared to pre-treatment values. Currently, the patient is still under treatment with CZP to prevent relapse of disease.

## Diagnostic Assessment

Before, during and after pregnancy the activity of CS was regularly monitored by CRP serum concentrations, along with clinical assessments including body weight, blood pressure and pulse rate, subjective hearing levels and tone audiometry, if necessary. In addition to the diagnostic parameters assessed above, blood smear with differential leukocyte count, and measurements of aspartate aminotransferase (AST), alanine aminotransferase (ALT), lactate dehydrogenase (LDH), creatine kinase (CK) and creatinine, together with urine analyses were performed at least quarterly. The fetal development was monitored by the patients’ gynecologists *via* ultrasound during every trimester of pregnancy. At gestational week 20 the fetus was screened for abnormalities and regular organ development. Additional Doppler imaging of placental blood flow was performed at specialized prenatal diagnostic centers twice during pregnancy. After childbirth the Appearance, Pulse, Grimace, Activity, Respiration (APGAR) score was assessed, and umbilical cord pH, neonatal weight, length and head circumference were measured. Developmental abnormalities were ruled out by regular pediatric screening tests, which are provided after birth and within the first years of life on a regular basis by the German health system.

## Discussion

Cogan’s syndrome is a systemic chronic inflammatory disease which can lead to severe functional visual and hearing impairments. It requires interdisciplinary clinical management and individualized treatment approaches by all specialists involved (2–4). Even more challenging is the adequate disease management in young female patients wishing conception or with ongoing pregnancy. Due to its systemic and inflammatory nature, pregnant patients with this complex vascular disease require unique attention and continuous clinical monitoring. The impact of a pregnancy itself on the course of CS is fairly unknown. Also, evidence-based treatment guidelines for CS are very limited with a paucity of randomized, double-blind trials comparing the efficacy of immunosuppressive agents. The current literature predominantly consists of retrospective chart analyses, small case series and single case reports. A recent review collected data on 87 adult CS patients and 17 pediatric patients (4) showing that methotrexate (MTX) was the most frequently used second-line therapy. Besides MTX there are reports on the successful use of azathioprine, cyclosporine, and cyclophosphamide as steroid-sparing immunosuppressive agents. However, immunosuppressive agents like MTX, cyclophosphamide and mycophenolate are contraindicated for their teratogenicity during pregnancy. More recently biological agents, especially TNF-α antagonists, and particularly infliximab, have shown favorable outcome (2–4).

Half of previously reported CS patients did not need any systemic immunosuppressive treatments throughout pregnancy, instead intermittent topical treatment with ophthalmic steroids was sufficient (10–13). Other pregnant patients in remission were successfully treated with hydroxychloroquine, or a combination therapy of either cyclosporine or intravenous immunoglobulin application (IVIG) with azathioprine and prednisolone (8, 9, 12). In contrast, our patients had the necessity of therapy escalation to anti-TNF therapy prior to conception because of disease activity and adverse effects. Accordingly, with its neglectable to minimal transplacental transfer in the third trimester of pregnancy (7), CZP was a particularly attractive second line therapy to avoid inflammatory flares and thereby protect mother and her unborn child. The European League Against Rheumatism (EULAR) recommends certolizumab as most favorable biological DMARD for the use throughout pregnancy and lactation in patients with inflammatory rheumatic diseases (5). Evidence for these recommendations is provided by two prospective pharmacokinetic studies, CRIB (NCT02019602) (7) and CRADLE (NCT02154425) (14). The CRIB study showed CZP plasma concentrations within the expected therapeutic range in 16 pregnant women at delivery, whereas no detectable or only minimal CZP serum concentrations were found in their newborns (7). The CRADLE study investigated CZP concentrations in human breast milk to estimate the daily dose of maternal CZP transferred to the infant by breast feeding (14). In most breast milk samples no measurable CZP was detectable, indicating no to minimal CZP transfer from maternal plasma to breast milk. Additionally, CZP absorption by the infants *via* breast milk is unlikely because of its low oral bioavailability. The mean age of all patients included in the CRIB and the CRADLE studies was approximately 30 years and therefore comparable to the CS patients reported here. Of relevance, none of the patients in these two studies had Cogan’s syndrome or any other form of systemic vasculitis. The most frequent indications for CZP treatment in CRIB/CRADLE were rheumatoid arthritis, followed by Crohn’s disease, psoriatic arthritis and axial spondyloarthritis (7, 14). Similar to our findings the gestational age and weight at birth of all 16 newborns in the CRIB study were within the expected range for healthy children (7). However, one limitation of our report is the low number of patients included, with CS being a very rare disease. Furthermore, we have no data on CZP pharmacokinetics in the three infants and their mothers. Though, we have no indication to expect results that are relevantly different to previously reported data in the CRIB and CRADLE studies. The serological and clinical improvement in both mothers before, during and after pregnancy reflects sufficient CZP serum concentrations in our patients. The uncomplicated pregnancies and the normal development of the children afterwards, without any infectious or other complications, support our hypothesis that the infants were exposed to relevant CZP doses during pregnancy or lactation period.

Another important finding is the fact that decreasing the CZP dose in patient A resulted in an increase of CRP serum concentrations. Cessation of CZP treatment more than a year after the second pregnancy, with the patient being in complete remission, led to a relapse of CS with typical clinical symptoms and high CRP concentrations.

Re-start of CZP lead to complete serological and clinical remission without relevant side effects, which demonstrates the long-term efficacy and safety of CZP in this patient. Also, the chronic disease course in this patient illustrates that CS may need long-term immunosuppressive treatment, occasionally even requiring TNFα-inhibitor administration.

To our knowledge this is the very first report on both the treatment with the monoclonal anti-TNFα-inhibitor CZP in CS and the first report on biological treatment during pregnancies in CS. In both of our patients CZP was effective and well tolerated before, during and after pregnancies. CRP values normalized, and tone audiograms showed stable or improved results compared to pre-CZP-treatment findings. All three pregnancies were without complications. All children were born healthy, at term, and developed regularly during follow-up.

In summary, CZP was shown to be effective and safe in the treatment of Cogan’s syndrome and should be considered as potential treatment option during pregnancy.

## Patient Perspective

Patient A: I agree to report my rare disease for a better medical understanding and improvement of treatment options in Cogan’s syndrome. I hope that this report enables physicians in Germany and other countries around the world to treat women with Cogan’s syndrome having a childbearing wish with a highly effective and, in my case, safe treatment. We are very happy to live a more or less normal life now as a family with two healthy and happy kids.

Patient B: I agree to report on my rare disease (Cogan’s syndrome), the pregnancy including the data of my child. I hope that this report will help and support other physicians and especially young female patients with Cogan’s syndrome.

## Data Availability Statement

The original data generated and analyzed for this study are included in the published article. Further inquiries can be directed to the corresponding author.

## Ethics Statement

Written informed consent was obtained from the individual for themselves and their child/children for the publication of any potentially identifiable images or data included in this article.

## Author Contributions

NV and CG wrote the manuscript. All authors were involved in direct patient care or acquisition of clinical data and contributed to the article and approved the submitted version.

## Conflict of Interest

NV, IJ, RV, and CG received payment (less than 5000€) from UCB pharmaceuticals for advisory boards, lectures, or travel grants within the last five years.

The remaining authors declare that the research was conducted in the absence of any commercial or financial relationships that could be construed as a potential conflict of interest.
